# Interactions of VMAT2 with CDCrel-1 and Parkin in Methamphetamine Neurotoxicity

**DOI:** 10.3390/ijms252313070

**Published:** 2024-12-05

**Authors:** Heli Chauhan, Nicholas J. Carruthers, Paul M. Stemmer, Bernard L. Schneider, Anna Moszczynska

**Affiliations:** 1 Department of Pharmaceutical Sciences, Eugene Applebaum College of Pharmacy and Health Sciences, Wayne State University, 259 Mack Ave., Detroit, MI 48201, USApmstemmer@wayne.edu (P.M.S.); 2 Institute of Environmental Health Sciences and Proteomics Core Facility, 540 East Canfield Ave., Detroit, MI 48202, USA; 3 Bioinformatics Core, Michigan Medicine, University of Michigan, NCRC Building 14, 2800 Plymouth Rd., Ann Arbor, MI 48109, USA; 4 Bertarelli Platform for Gene Therapy, École Polytechnique Fédérale de Lausanne, School of Life Sciences, Ch. Des Mines 9, CH-1202 Geneva, Switzerland

**Keywords:** methamphetamine neurotoxicity, vesicular monoamine transporter-2, parkin, CDCrel-1, exocytosis/endocytosis cycle

## Abstract

In recent years, methamphetamine (METH) misuse in the US has been rapidly increasing, and there is no FDA-approved pharmacotherapy for METH use disorder (MUD). In addition to being dependent on the drug, people with MUD develop a variety of neurological problems related to the toxicity of this drug. A variety of molecular mechanisms underlying METH neurotoxicity has been identified, including the dysfunction of the neuroprotective protein parkin. However, it is not known whether parkin loss of function within striatal dopaminergic (DAergic) terminals translates into decreased DA storage capacity. This study examined the relationship between parkin, its substrate cell division cycle related-1 (CDCrel-1) associated with synaptic vesicles, and vesicular monoamine transporter-2 (VMAT2) responsible for packaging DA in an in vivo model of METH neurotoxicity. To assess the individual differences in response to METH’s neurotoxic effects, a large group of male Sprague Dawley rats were treated with binge METH or saline and sacrificed 1 h or 24 h later. This study is the first to show that CDCrel-1 interacts with VMAT2 in the rat striatum and that binge METH can alter this interaction as well as the levels and subcellular localization of CDCrel-1. The proteomic analysis of VMAT-2-associated proteins revealed the upregulation of several proteins involved in the exocytosis/endocytosis cycle and responses to stress. The results suggest that DAergic neurons are engaged in counteracting METH-induced toxic effects, including attempts to increase endocytosis and autophagy at 1 h after the METH binge, with the responses varying widely between individual rats. Studying CDCrel-1, VMAT2, and other proteins in large groups of outbred rats can help define individual genetic and molecular differences in responses to METH neurotoxicity, which, in turn, may aid treating humans suffering from MUD and its neurological consequences.

## 1. Introduction

Methamphetamine (METH) is a highly addictive and powerful central nervous system psychostimulant that induces a feeling of intense euphoria. Therefore, METH misuse is a worldwide health problem. In the United States, there are close to 2,000,000 people who misuse METH, and deaths from METH overdose are rapidly rising [1,2]. METH overdose is not the only danger to health. METH is highly neurotoxic, and its misuse causes a variety of serious neurological problems and is associated with a risk of developing Parkinson’s disease [3,4,5]. A deficiency in dopamine (DA) within the nigrostriatal DA pathway is believed to underlie this risk [6]. Despite numerous clinical trials conducted to date, there is no FDA-approved medication for METH use disorder (MUD) or the neurological problems associated with the disorder. Such medication is particularly needed for people with severe MUD (people who use METH heavily) as they have the most difficulty quitting METH use, are at high risk of overdosing on the drug, and suffer the most from the neurological consequences of METH neurotoxicity.

In experimental animals, high METH doses (4 × 5–20 mg/kg or single > 30 mg/kg) are neurotoxic to DAergic and serotonergic nerve terminals in selective brain areas, with the dorsal striatum being the most affected [7,8]. At the molecular level, METH neurotoxicity to DAergic and serotonergic terminals in experimental animals is manifested by persistent (long-lasting after METH cessation) reductions in DAergic and serotonergic markers (DA, serotonin and their metabolites, DA and serotonin transporters, vesicular monoamine transporter-2 (VMAT2), tyrosine hydroxylase, and tryptophan hydroxylase) [9,10,11,12], microglial activation and gliosis [13,14], as well as morphological and structural abnormalities such as brain edema, increased blood–brain barrier permeability, swollen neuronal axons, and damaged myelin [15,16,17]. The METH-mediated neurotoxic effects and their manifestation in individuals addicted to METH are similar to those observed in experimental animals; however, the actual degeneration of DAergic and five serotonergic nerve terminals, or cell bodies, has been questioned [6]. Nevertheless, many human METH users suffer from persistent DAergic and serotonergic deficits as well as from brain structural and metabolic abnormalities in the same brain areas as those affected by METH in experimental animals, suggesting the functional dysregulation of these areas (reviewed in [7,8,18]).

Our previous studies showed that parkin, a neuroprotective ubiquitin-protein ligase, protected DAergic terminals in rat dorsal striatum from METH neurotoxicity [19] and might be a potential new drug target in severe MUD [20]. Others have shown an essential role of VMAT2 in METH neurotoxicity and MUD [21,22,23,24]. It is not known whether there is an interaction between parkin and VMAT2 in METH neurotoxicity.

The VMAT2 is a proton pump localized on synaptic monoaminergic storage vesicles and controls intracellular DA levels, transporting DA from the cytoplasm into the vesicles in exchange for protons [25,26]. METH reverses VMAT2 function and disrupts the proton gradient of the vesicles, mechanisms through which it stimulates the efflux of DA from the storage vesicles to the cytoplasm [27,28]. The release of DA by METH is a first step in METH neurotoxicity as DA rapidly autoxidizes in the cytoplasm, leading to oxidative stress and damage to proteins, lipids, and organelles within DAergic terminals [29]. In addition, neurotoxic METH doses rapidly redistribute rat striatal VMAT2 immunoreactivity from synaptic vesicle-enriched, nonmembrane subcellular fractions to a location not retained in the preparation of the synaptosomes while leaving VMAT2 membrane expression unchanged [30] or increased [31]. This impaired recycling of synaptic VMAT2 vesicles from the plasma membrane to the cytosol suggested a possibility that high-dose METH might render membrane VMAT2 vesicles trapped at the plasma membrane and nonfunctional while removing other VMAT2 vesicles from DAergic terminals via retrograde axonal transport. These changes render DAergic terminals with a decreased ability to sequester DA, thus increasing the pro-oxidant effects of the neurotransmitter.

Parkin is an E3 ligase in the ubiquitin-proteasome system that protects DA neurons from diverse cellular insults, including METH- and DA-induced oxidative stress [19,32]. This protection may be partially exerted via the regulation of DAergic neurotransmission. Thus, parkin has been found to regulate DA transporter trafficking between the plasma membrane and cytosol in vitro [33]. It is not known whether parkin has a similar effect on VMAT2 under physiological or neurotoxic conditions in vivo. The available literature data suggest that parkin may regulate VMAT2 vesicle trafficking via parkin substrates involved in the exocytosis/endocytosis cycle, namely, α-synuclein [34], α- and β-tubulin [35], synaptotagmins IV and 11 [36,37], synphilin-1 [38], endophilin, dynamin, synaptojanin-1 [39], and CDCrel-1 (septin 5) [40,41]. Without parkin, these substrates would abnormally accumulate. Song and colleagues reported that parkin modulated the endo-lysosomal pathway in cultured cells [42]. Feng and colleagues reported that parkin stabilized microtubules (MTs) and facilitated the transport of misfolded proteins along the MTs for degradation [35]. It is not known whether the METH neurotoxicity-induced deficit in striatal parkin levels that our group demonstrated in [12] mediates the redistribution of VMAT2 vesicles away from striatal terminals observed by the aforementioned previous studies [30,31].

CDCrel-1 is a filament-forming protein involved in the inhibition of the exocytotic process [43,44]. The overexpression of CDCrel-1 produces DA neuron-specific cell death [45], which can be prevented by increasing parkin levels [46]. Both parkin and VMAT2 were shown to be oxidatively damaged and decreased after the administration of high METH doses [12,47,48], while the effects of METH on CDCrel-1 have not been studied. Moreover, little is known about the interactions between parkin, VMAT2, and CDCrel-1 under METH-induced neurotoxic conditions. Knowledge of the effects of neurotoxic METH doses on endocytic and exocytic proteins is also limited. We tested the hypothesis that METH-induced parkin deficit increases CDCrel-1 levels in rat striatal neuronal terminals, which, in turn, alters the localization of VMAT2 vesicles and decreases DA storage within the terminals.

## 2. Results

### 2.1. The 4 × 8 mg/kg METH Binge Elicits Variable Thermal Responses in Wild-Type Rats

**Figure 1:**
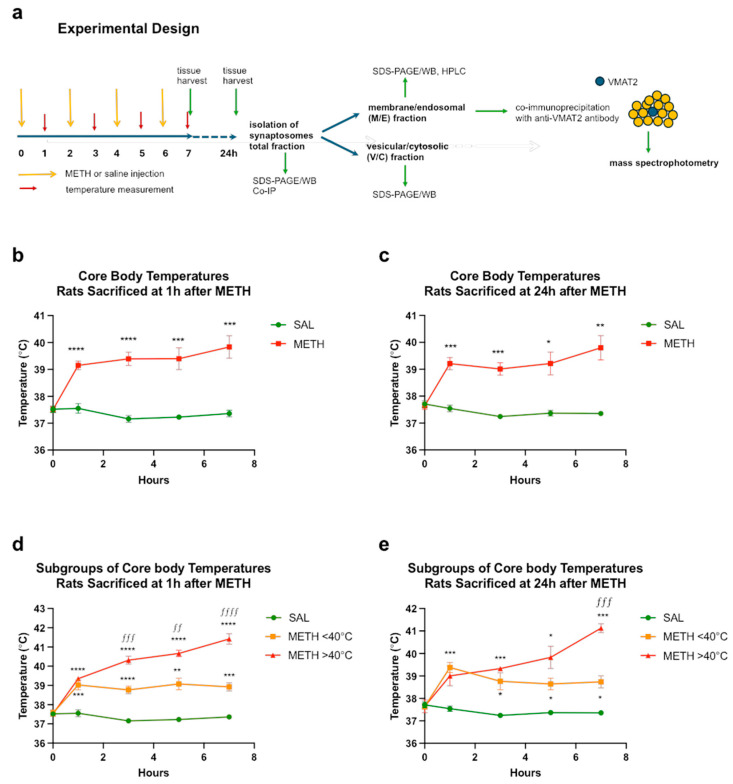
Experimental design and assessment of METH neurotoxicity. (**a**) A total of 8.0 mg/kg free base METH or saline (1 mL/kg) was administered to rats every 2 h in four successive intraperitoneal (i.p.) injections. Core body temperatures (°C) were measured before the first METH or saline injection and 1 h after each METH or saline injection. The rats were sacrificed 1 h or 24 h after the last injection of METH or saline. Stratal synaptosomes were isolated, separated into total, membrane/endosomal, and vesicular/cytosolic fractions, and analyzed. Proteomic analysis was performed on VMAT2-associated proteins coimmunoprecipitated from membrane/endosomal fractions. (**b**,**c**) Core body temperatures of rats euthanized at 1 h (**b**) or 24 h (**c**) after METH or saline. (**d**,**e**) The METH-treated rats were divided into two groups depending on the severity of hyperthermia: those with high hyperthermia and those with low hyperthermia (average of 4 temperature readings >40 °C and <40 °C, respectively) and euthanized at 1 h (**d**) or 24 h (**e**) after METH or saline. Significant differences between saline and METH rats: * *p* < 0.05, ** *p* < 0.01, *** *p* < 0.001, and **** *p* < 0.0001. Significant differences between HH and LH rats: *^ƒƒ^ p* < 0.01, *^ƒƒƒ^ p* < 0.001, and *^ƒƒƒƒ^ p* < 0.0001. Values expressed as mean ± SEM. Abbreviations: METH, methamphetamine; SAL, saline.

In experimental animals, high doses of METH are known to cause hyperthermia, which is one of the mediators and indicators of METH neurotoxicity [29,49]. To confirm that METH binge-induced hyperthermia in the rats, their core body temperature was measured at 1 h after each METH or saline injection. Significant increases in core body temperatures were observed in all METH-treated wild-type (WT) rats compared to saline controls (two-way RT ANOVA followed by the Sidak post hoc test) (Figure 1b,c). All the WT rats reached 39 °C at least once. There was a significant main effect of the treatment (METH vs. saline) in both experimental cohorts, i.e., rats sacrificed at 1 h and those sacrificed at 24 h after the last METH injection (1 h cohort: treatment *F*(1,28) = 52.6, *p* < 0.0001, time (*F*(2.42,67.8) = 10.7, *p* < 0.0001, and treatment × time interaction (*F*(4,112) = 16.2, *p* < 0.0001; 24 h cohort: treatment *F*(1,16) = 60.6, *p* < 0.0001), time (*F*(2.42,37.6) = 5.99, *p* < 0.01, and treatment × time interaction (*F*(4,62) = 11.6, *p* < 0.0001)). It was observed that despite the same METH regimen, the thermal responses to METH were highly variable. The METH-treated rats could be divided into two groups depending on the severity of hyperthermia: those with high hyperthermia (HH) and those with low hyperthermia (LH) (Figure 1d,e). Significant differences in core body temperatures were observed between the saline and METH treatments as well as between the subgroups (1 h cohort: treatment *F*(2,27) = 106, *p* < 0.0001, time (*F*(3.15,83.5) = 53.1, *p* < 0.0001, and treatment × time interaction (*F*(8,106) = 29.3, *p* < 0.0001; 24 h cohort: treatment *F*(2,15) = 61.1, *p* < 0.0001), time (*F*(3.31,49.7) = 23.5, *p* < 0.0001, and treatment × time interaction (*F*(8,60) = 18.3, *p* < 0.0001)). Areas under the curve (AUC) were calculated for all METH-treated rats.

### 2.2. The Effects of the 4 × 8 mg/kg METH Binge on α-Tubulin and β-Actin Immunoreactivity in Striatal Synaptosomal Fractions

**Figure 2:**
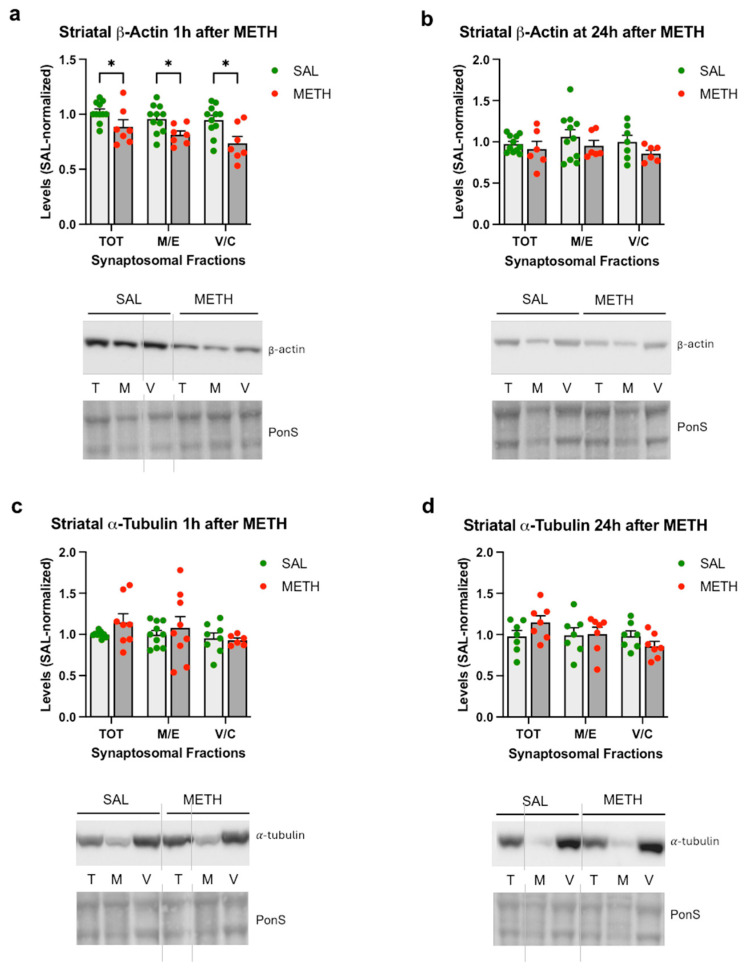
The effects of the 4 × 8 mg/kg METH binge on α-tubulin and β-actin immunoreactivity in striatal synaptosomal fractions. Immunoreactivity of β-actin (**a**,**b**) or α-tubulin (**c**,**d**) in the total (TOT or T), membrane/endosomal (M/E or M), and vesicular/cytosolic (V/C or C) synaptosomal fractions of the striatum in rats that were euthanized at 1 h (**a**,**c**) or 24 h (**b**,**d**) after the last dose of saline or METH. * *p* < 0.05, *n* = 7–11. Values are expressed as mean ± SEM. Vertical grey lines show where the blot was cut for rearrangement. Abbreviations: METH, methamphetamine; SAL, saline; PonS, Ponceau S.

There is surprisingly little literature on the effect of METH on the cytoskeleton. The available data suggest that METH can disorganize both MTs and actin filaments [50]. Therefore, we compared β-actin and α-tubulin immunoreactivities in all striatal synaptosomal fractions at 1 h and 24 h after the last dose of METH vs. saline. β-Actin immunoreactivity was significantly decreased in all three synaptosomal fractions at 1 h after METH (total fraction: −17%, membrane/endosomal fraction: −19%, and vesicular/cytosolic fraction: −26%, *p* < 0.05, multiple unpaired *t*-tests with Holm–Sidak correction for multiple comparisons, *n* = 7–11) (Figure 2a). No significant changes were detected in β-actin immunoreactivity at 24 h after METH (Figure 2b). No statistically significant changes were detected at 1 h or 24 h after METH in the levels of α-tubulin; however, individual variability in its immunoreactivity was observed in the total and membrane/endosomal synaptosomal fractions from binge METH-treated rats at the 1 h time point (Figure 2c,d), thus suggesting that METH influenced α-tubulin levels differently in different rats. Consequently, to control for METH effects on the cytoskeleton, we used total protein levels (measured by Ponceau S dye) as a loading control in the subsequent quantification of immunoreactivity on Western blots.

### 2.3. The Effects of the 4 × 8 mg/kg METH Binge on Parkin Immunoreactivity in Striatal Synaptosomal Fractions

**Figure 3:**
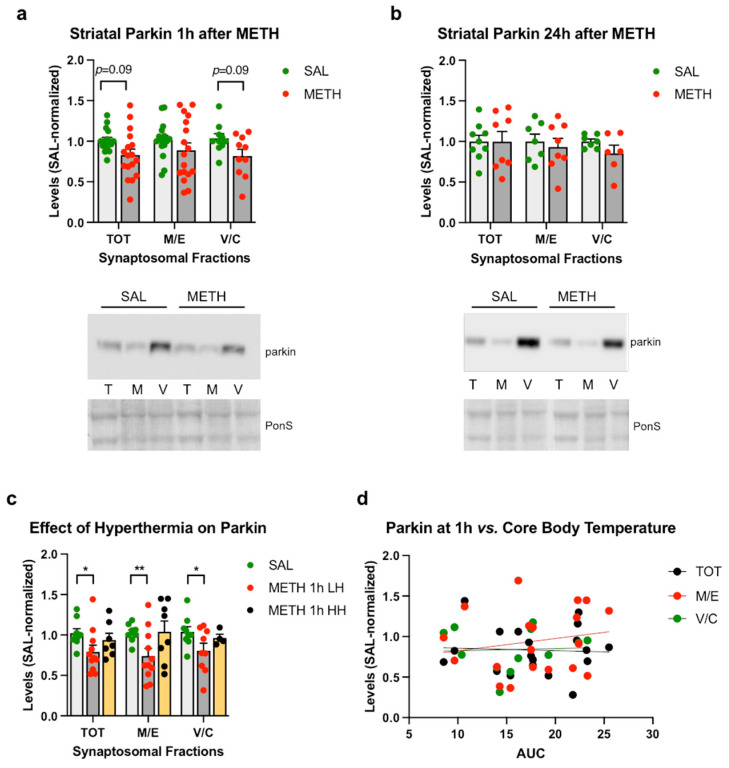
The effects of the 4 × 8 mg/kg METH binge on parkin immunoreactivity in striatal synaptosomal fractions. Immunoreactivity of parkin in the total (TOT or T), membrane/endosomal (M/E or M), and vesicular/cytosolic (V/C or C) synaptosomal fractions of the striatum in rats euthanized at (**a**) 1 h or (**b**) 24 h. (**c**) Immunoreactivity of parkin in striatal synaptosomal fractions in high-hyperthermia (HH) and low-hyperthermia (LH) rats. (**d**) Correlations of parkin immunoreactivity in synaptosomal fractions with core body temperature (area under the curve AUC) of the rats sacrificed 1 h after the last METH dose. * *p* < 0.05, ** *p* < 0.01, *n* = 10–17. Values are expressed as mean ± SEM. Abbreviations: METH, methamphetamine; SAL, saline; PonS, Ponceau S.

We previously determined that 4 × 10 mg/kg METH decreased parkin immunoreactivity in the total striatal synaptosomal fraction at 1 h and 24 h after the last dose of the drug, with parkin immunoreactivity returning to the baseline by 48 h [12]. In this study, we used 4 × 8 mg/kg METH to decrease the mortality rate. The 4 × 8 mg/kg dose did not significantly decrease parkin immunoreactivity in synaptosomal fractions compared to the saline controls; however, a trend toward statistical significance was observed in the total and vesicular/cytosolic synaptosomal fractions (−18% and −20%, respectively, *p* = 0.09, unpaired multiple *t*-tests with Holm–Sidak correction for multiple comparisons, *n* = 10–17) at 1 h after the last METH dose (Figure 3a). No significant changes in parkin levels (or trends toward statistical significance) were detected at 24 h after METH compared to the saline controls (Figure 3b). As with core body temperature, high variability in parkin immunoreactivity in METH-treated rats was observed at both time points (Figure 3a,b). When the HH and LH rats from the 1 h group were assessed separately, a significant deficit in parkin immunoreactivity was detected in the LH rats in all synaptosomal fractions (two-way ANOVA followed by the Holm–Sidak post hoc test; main effect of treatment: *F*(1,52) = 15.74, *p* < 0.001; total fraction: −23%, *p* < 0.05; membrane/endosomal fraction: −28%, *p* < 0.01; vesicular/cytosolic fraction: −23%, *p* < 0.05), with HH rats displaying unchanged parkin immunoreactivity (Figure 3d). However, there was no significant correlation between core body temperature and parkin immunoreactivity in synaptosomal fractions, indicating an influence of additional factor(s) on parkin immunoreactivity in the METH-treated rats. In METH neurotoxicity-resistant cerebellum, a significant increase in synaptosomal parkin was detected compared to the saline controls (Supplementary Figure S1).

### 2.4. The Effects of the 4 × 8 mg/kg METH Binge on VMAT2 Immunoreactivity in Striatal Synaptosomal Fractions

**Figure 4:**
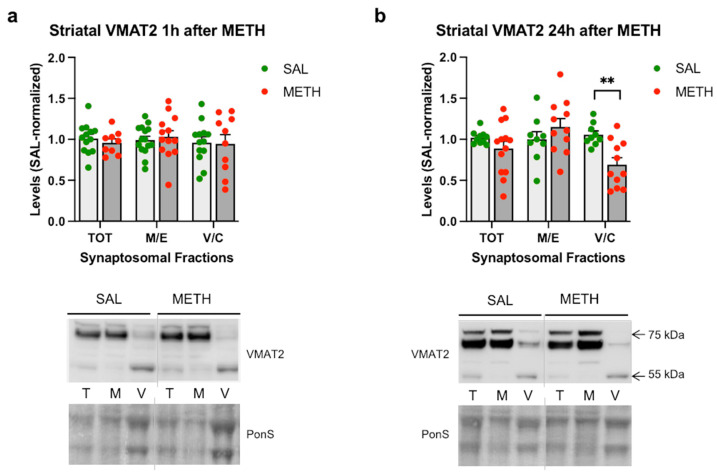

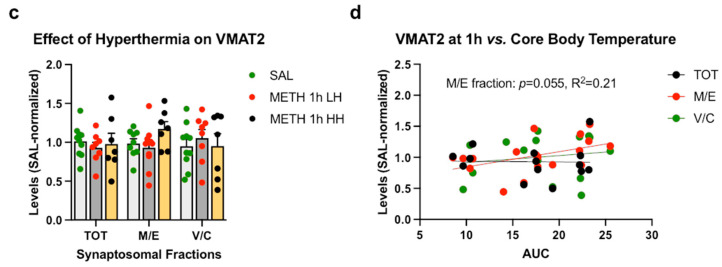
The effects of the 4 × 8 mg/kg METH binge on VMAT2 immunoreactivity in striatal synaptosomal fractions. Immunoreactivity of VMAT2 in the total (TOT or T), membrane/endosomal (M/E or M), and vesicular/cytosolic (V/C or C) synaptosomal fractions of the striatum in rats euthanized at (**a**) 1 h or (**b**) 24 h. ** *p* < 0.01, *n* = 9–11. (**c**) Immunoreactivity of VMAT2 in striatal synaptosomal fractions in high-hyperthermia (HH) and low-hyperthermia (LH) rats. (**d**) Correlations of VMAT2 immunoreactivity in synaptosomal fractions with core body temperature (area under the curve, AUC) of rats sacrificed 1 h after the last METH dose. A strong trend toward statistical significance was detected in the membrane/endosomal fraction (*p* = 0.055). Values are expressed as mean ± SEM. Vertical grey lines show where the blot was cut for rearrangement. Abbreviations: METH, methamphetamine; SAL, saline; PonS, Ponceau S.

Of the three isoforms of VMAT2, our anti-VMAT2 antibody easily identified the partially glycosylated form (~55 kDa) and the glycosylated form (68–75 kDa) of the protein; the non-glycosylated VMAT2 (45 kDa) was detected at low levels (Figure 4). At 1 h after METH, we did not detect significant changes in VMAT2 immunoreactivity in synaptosomal fractions as compared to the saline controls (Figure 4a). At 24 h after METH, a significant decrease in VMAT2 immunoreactivity was detected in the vesicular/cytosolic fraction (−34%, *p* < 0.05, unpaired multiple *t*-tests with Holm–Sidak correction for multiple comparisons, *n* = 9–11) in METH-treated rats relative to the saline-treated rats (Figure 4b). As with individual parkin levels, high variability in individual synaptosomal VMAT2 immunoreactivity was observed after the METH binge at both time points, 1 h and 24 h. Subgrouping VMAT2 values from the 1 h METH group according to the levels of hyperthermia did not produce significant changes in VMAT2 immunoreactivity as compared to the corresponding saline group (Figure 4c). There was no significant correlation between core body temperature and VMAT2 immunoreactivity in the vesicular/membrane fraction in the METH-treated rats (Figure 4d); however, the correlation between core body temperature and membrane/endosomal VMAT2 displayed a strong trend toward statistical significance (*p* = 0.055, R^2^ = 0.21, Pearson’s correlation test).

### 2.5. Parkin Does Not Affect VMAT2 Immunoreactivity in Striatal Synaptosomal Fractions

**Figure 5:**
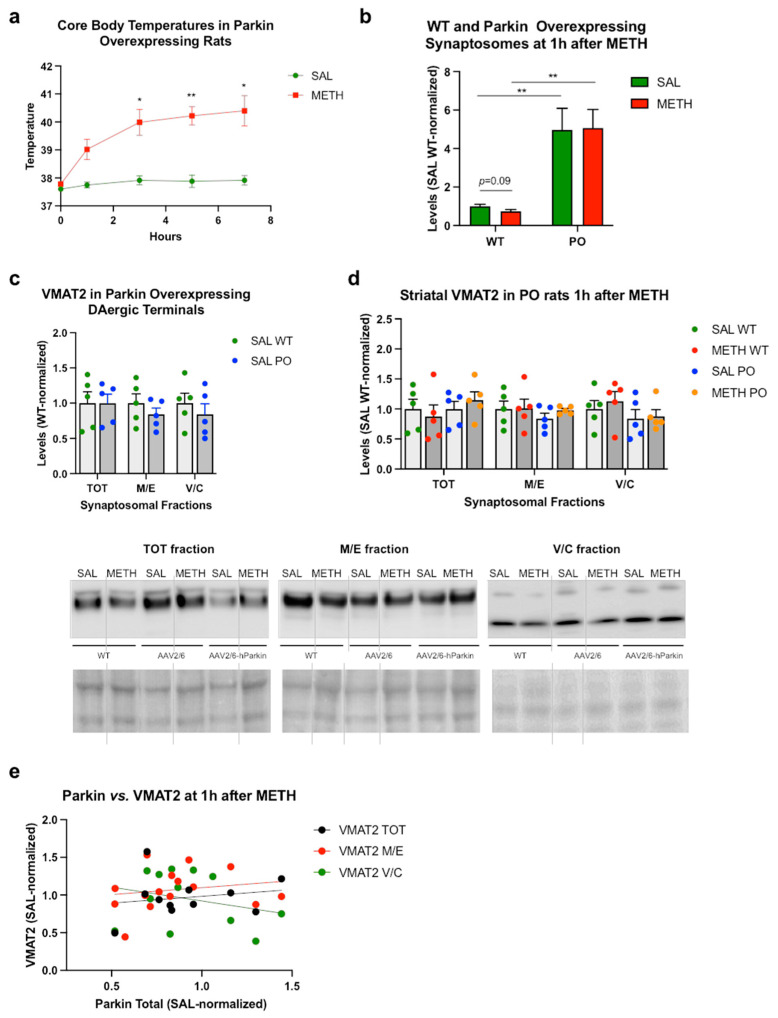
Parkin does not affect VMAT2 immunoreactivity in striatal synaptosomal fractions. (**a**) Core body temperatures (°C) of the wild-type rats and rats overexpressing parkin in the nigrostriatal dopamine pathway. * *p* < 0.05, ** *p* < 0.01. (**b**) Immunoreactivity of parkin in the total synaptosomal fraction of the striatum in rats euthanized at 1 h after METH or saline. ** *p* < 0.01 *n* = 5. Parkin overexpression was about 5-fold in both treatment groups. METH did not significantly alter parkin immunoreactivity in the wild-type (*p* = 0.09) or parkin-overexpressing (*p* > 0.1) striatal synaptosomes at 1 h after the treatment. (**c**) Immunoreactivity of VMAT2 in the total (TOT), membrane/endosomal (M/E), and vesicular/cytosolic (V/C) synaptosomal fractions in the striatum from wild-type and parkin-overexpressing saline-treated rats. (**d**) Immunoreactivity of VMAT2 in striatal synaptosomal fractions in the wild-type and parkin-overexpressing rats treated with METH or saline at 1 h after the treatment. (**e**) Correlations of VMAT2 immunoreactivity in synaptosomal fractions with total synaptosomal parkin immunoreactivity. Values are expressed as mean ± SEM. Vertical grey lines show where the blot was cut for rearrangement. Abbreviations: AAV2/6, adeno-associated viral vector 2/6; METH, methamphetamine; SAL, saline; PO, parkin-overexpressing; WT, wild-type; PonS, Ponceau S; AAV2/6-parkin, parkin-encoding AAV2/6; AAV6, non-coding AAV2/6.

Since parkin binds with MTs and actin filaments [51,52] and the available evidence suggests its involvement in the exocytosis/endocytosis cycle, parkin may play a role in the intracellular trafficking of VMAT2-containing vesicles. To test this hypothesis, we assessed VMAT2 immunoreactivity in striatal synaptosomes from rats overexpressing parkin in the nigrostriatal DA pathway. Parkin overexpressed about 5-fold in all synaptosomal fractions (Figures S2b and S3). The binge METH-treated parkin-overexpressing rats displayed a similar profile of thermal response to METH as the WT rats did (treatment *F*(1,10) = 102, *p* < 0.0001, time (*F*(1.77,17.7) = 18.1, *p* < 0.01, and treatment × time interaction (*F*(4,40) = 4.92, *p* < 0.01, one-way RT ANOVA followed by the Sidak post hoc test) (Figure 5a). As in the first METH experiment, binge METH treatment caused a small decrease in parkin immunoreactivity in WT rat striatum that displayed a trend toward statistical significance when analyzed by Student’s *t*-test (−26%, *p* = 0.09, *n* = 5) (Figure 4b; a representative blot is presented in Supplementary Figure S3). The trend was lost when WT and parkin overexpression data were analyzed using two-way ANOVA. METH treatment did not significantly change parkin immunoreactivity in parkin-overexpressing rats (treatment and treatment × parkin levels *p* > 0.05; parkin levels *F*(1,16) = 31.4, *p* < 0.0001) (Figure 5b). The overexpression of parkin did not affect VMAT2 immunoreactivity in any synaptosomal fraction in the saline-treated rats (*p* > 0.05, *n* = 5, multiple unpaired *t*-tests with Holm–Sidak correction for multiple comparisons) (Figure 5c). Binge METH did not decrease VMAT2 immunoreactivity in striatal synaptosomal fractions from the WT or parkin-overexpressing rats at 1 h after the last METH injection (*p* > 0.02, *n* = 5, two-way ANOVA with the Holm–Sidak post hoc test) (Figure 5d). There was no significant correlation between total parkin immunoreactivity and total, membrane/endosomal, or vesicular/cytosolic immunoreactivity of VMAT2 in METH-treated rats at 1 h after the treatment (*p* > 0.05, Pearson’s correlation test) (Figure 5e).

### 2.6. The Effects of the 4 × 8 mg/kg METH Binge on CDCrel-1 Immunoreactivity in Striatal Synaptosomal Fractions 

**Figure 6:**
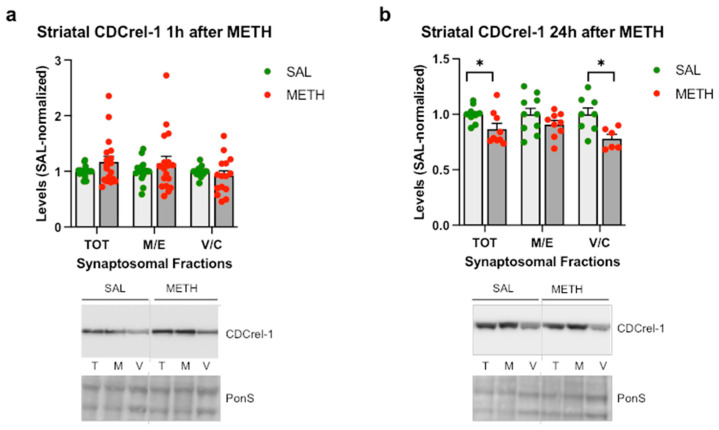

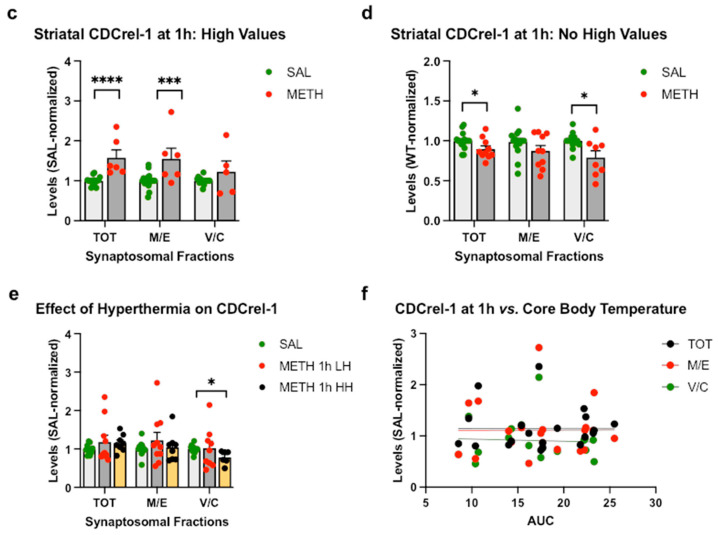
The effects of the 4 × 8 mg/kg METH binge on CDCrel-1 immunoreactivity in striatal synaptosomal fractions. Immunoreactivity of CDCrel-1 in the total (TOT or T), membrane/endosomal (M/E or M), and vesicular/cytosolic (V/C or C) synaptosomal fractions of the striatum in rats euthanized at (**a**) 1 h or (**b**) 24 h. * *p* < 0.05, *n* = 9–10. (**c**,**d**) Immunoreactivity of CDCrel-1 in striatal synaptosomal fractions separated into two subgroups based on individual variability to METH. * *p* < 0.05, *** *p* < 0.001, **** *p* < 0.0001, *n* = 6–13. (**e**) Immunoreactivity of CDCrel-1 in striatal synaptosomal fractions in high-hyperthermia (HH) and low-hyperthermia (LH) rats. * *p* < 0.05, *n* = 6–10. (**f**) Correlations of CDCrel-1 immunoreactivity in synaptosomal fractions with core body temperature (area under the curve, AUC) of the wild-type rats sacrificed 1 h after the last METH dose. Values are expressed as mean ± SEM. Vertical grey lines show where the blot was cut for rearrangement. Abbreviations: METH, methamphetamine; SAL, saline; PonS, Ponceau S.

To determine the effect of the METH binge on CDCrel-1 levels in rat striatal synaptosomes, we assessed CDCrel-1 immunoreactivity in the WT rats treated with saline or 4 × 8 mg/kg binge METH. As compared to the saline controls, no significant changes in CDCrel-1 immunoreactivity in striatal synaptosomal fractions were detected in the WT rats at 1 h after the last METH injection (Figure 6a), whereas a significant decrease in CDCrel-1 immunoreactivity was detected in the total and vesicular/cytosolic fractions at 24 h after METH as compared to the saline controls (−13% and −22%, respectively, *p* < 0.05, *n* = 9–10, multiple unpaired *t*-tests with Holm–Sidak correction for multiple comparisons) (Figure 6b). As with other measured indices, a wide range of individual responses to the drug was observed at 1 h, with some rats having very high CDCrel-1 immunoreactivity (Figure 6a). After dividing the METH group into two subgroups (group 1 with total synaptosomal CDCrel-1 immunoreactivity at a >120% increase and group 2 with total synaptosomal CDCrel-1 immunoreactivity at a <120% increase, with no change, or at a decrease), significant METH-induced increases in the total and membrane/endosomal fractions in group 1 were observed (+58%, *p* < 0.0001 and +55%, *p* < 0.001, *n* = 6–13, multiple unpaired *t*-tests with Holm–Sidak correction for multiple comparisons) and significant METH-induced decreases in the total and vesicular/cytosolic fractions in group 2 emerged (−10% and −21%, respectively, *p* < 0.05, *n* = 11–13, multiple unpaired *t*-tests with Holm–Sidak correction for multiple comparisons) (Figure 6c,d). CDCrel-1 immunoreactivity was not significantly changed or markedly varied in the control cerebellum (Supplementary Figure S1). After dividing the METH rats according to their thermal responses to METH (low or high hyperthermia: LH or HH), a small statistically significant decrease in CDCrel-1 immunoreactivity was observed in the vesicular/cytosolic fraction in the HH rats as compared to the saline controls (−22%, *p* < 0.05, multiple unpaired *t*-tests with Holm–Sidak correction for multiple comparisons) (Figure 6e). There was no significant correlation between the core body temperature and CDCrel-1 immunoreactivity in synaptosomal fractions, suggesting that core body temperature does not have marked influence on CDCrel-1 levels in striatal synaptosomes (*p* > 0.05, Pearson’s correlation test) (Figure 6f).

### 2.7. CDCrel-1 Interactions with Parkin in Striatal Synaptosomal Fractions 

**Figure 7:**
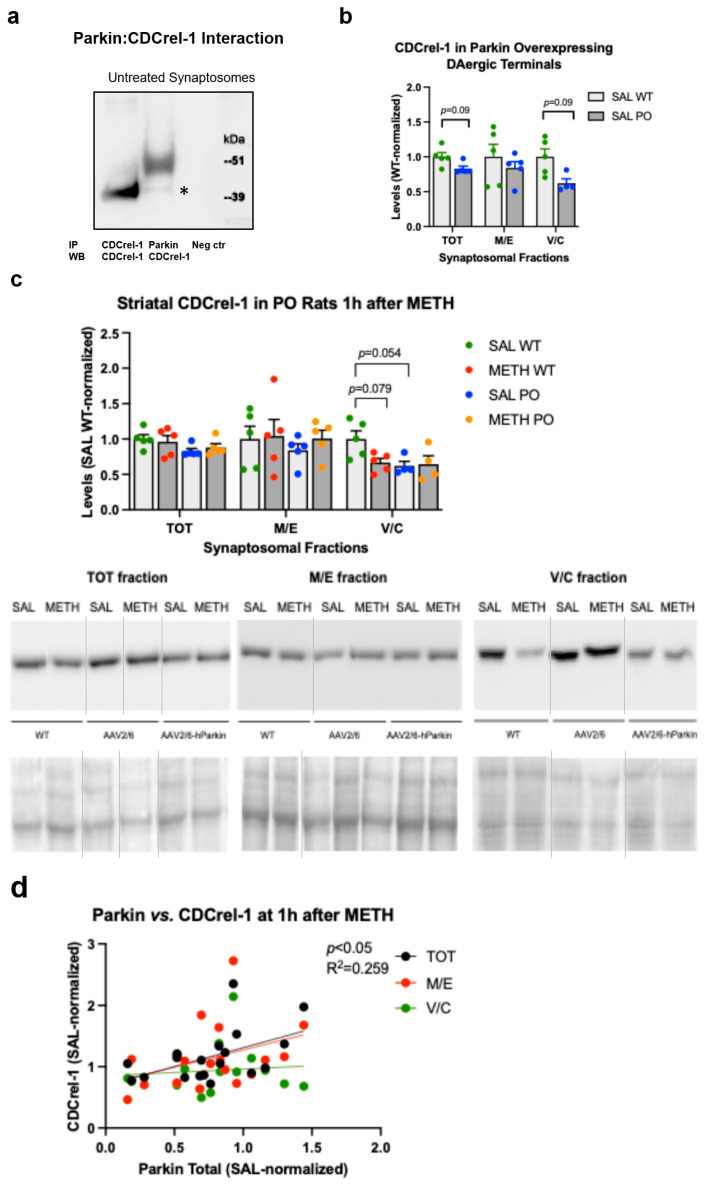
CDCrel-1 interactions with parkin in striatal synaptosomal fractions. (**a**) Anti-parkin antibody immunoprecipitated CDCrel-1 from untreated striatal synaptosomes (asterisk). (**b**) There was a weak trend toward statistical significance for the overexpression of parkin in the nigrostriatal dopamine pathway decreasing CDCrel-1 levels in the total and vesicular/cytosolic synaptosomal fractions (*p* = 0.09, *n* = 5). (**c**) Trends toward statistical significance for parkin and METH decreasing CDCrel-1 immunoreactivity in the wild-type rats at 1 h after the last dose of the drug were detected (*p* = 0.054 and *p* = 0.079, respectively, *n* = 5). METH treatment did not decrease CDcrel-1 levels in parkin-overexpressing rats. (**d**) Correlations of CDCrel-1 immunoreactivity in synaptosomal fractions with parkin immunoreactivity of the wild-type rats sacrificed 1 h after the last METH dose. A statistically significant positive correlation was found in the total synaptosomal fraction. Values are expressed as mean ± SEM. Abbreviations: METH, methamphetamine; SAL, saline; PonS, Ponceau S; AAV2/6-parkin, parkin-encoding AAV2/6; AAV6, non-coding AAV2/6; IP, immunoprecipitation; WB, Western blotting.

In vitro, parkin regulates the levels of CDCrel-1, a protein that regulates cytoskeleton organization and inhibits exocytosis [41,43]. CDCrel-1 also induces the neurodegeneration of DA neurons when overexpressed in the nigrostriatal DA pathway [45]. Consequently, the METH-induced deficit in parkin could have increased CDCrel-1 levels and contributed to METH-induced neurodegeneration of DAergic terminals in the rat striatum, at least in some of the METH-treated rats. To determine the effect of parkin on CDCrel-1 levels in rat striatal synaptosomes, we first performed a coimmunoprecipitation experiment. As shown in Figure 7a, the anti-parkin antibody coimmunoprecipitated a small amount of CDCrel-1 with parkin in striatal synaptosomes from the WT rats. Next, we assessed CDCrel-1 immunoreactivity in parkin-overexpressing rats. The overexpression of parkin in the nigrostriatal DA pathway resulted in trends toward statistical significance in the total and vesicular/cytosolic fractions in METH-naïve rats (−18%, *p* = 0.09 and −43%, *p* = 0.09, *n* = 5, multiple unpaired *t*-tests with Holm–Sidak correction for multiple comparisons) (Figure 7b). CDCrel-1 immunoreactivity showed a trend toward a statistically significant decrease in the vesicular/cytosolic fraction after binge METH administration in the WT rats (−33%, *p* = 0.079, *n* = 5, multiple unpaired *t*-tests with Holm–Sidak correction for multiple comparisons) (Figure 7b). The trend towards statistical significance was lost when all treatment data were analyzed using two-way ANOVA (Figure 7c). No METH-induced high CDCrel-1 immunoreactivity was observed in this experiment, likely because of the smaller sample size as compared to the first experiment with the WT rats. These results agree with those shown in Figure 7d (a subgroup with no high CDCrel-1 values). As aforementioned, the METH-naïve parkin-overexpressing rats displayed a 33% decrease in vesicular/cytosolic CDCrel-1 as compared to the WT rats. The administration of the METH binge did not further decrease CDCrel-1 immunoreactivity in this fraction in the rats overexpressing parkin in the nigrostriatal DA pathway (PO rats) (Figure 7c). The correlation of parkin immunoreactivity with CDCrel-1 immunoreactivity was positive and statistically significant in the total synaptosomal fraction at 1 h after METH (*p* < 0.05, R^2^ = 0.259, Pearson’s correlation test) (Figure 7d). 

### 2.8. CDCrel-1 Interactions with VMAT2 in Striatal Synaptosomes 

**Figure 8:**
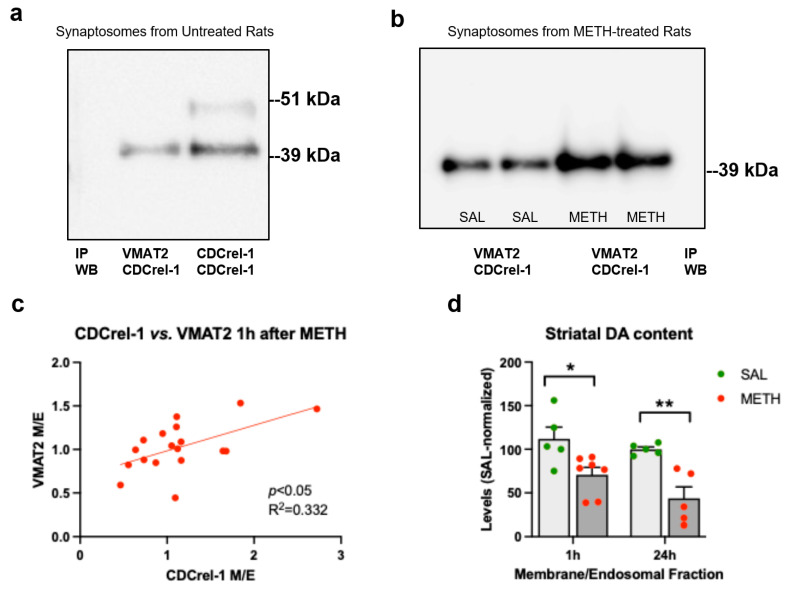

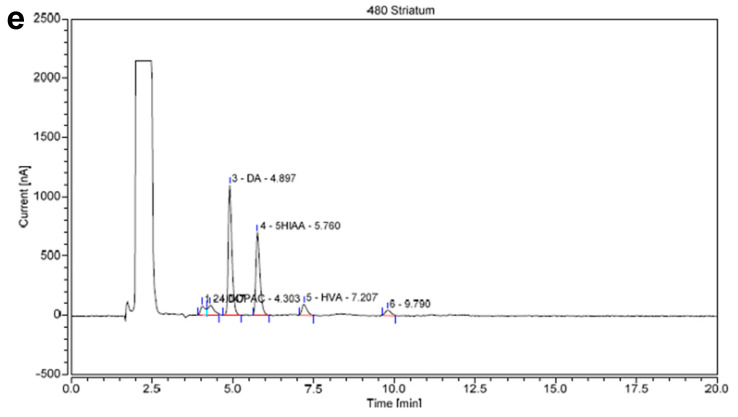
CDCrel-1 interactions with VMAT2 in striatal synaptosomes. (**a**) Anti-VMAT2 antibody immunoprecipitated CDCrel-1 from untreated striatal synaptosomes. (**b**) METH treatment increased the amount of immunoprecipitated CDCrel-1 at 1 h after the last dose of the drug. (**c**) There was a significant positive correlation between CDCrel-1 and VMAT2 immunoreactivity in the membrane/endosomal fraction of striatal synaptosomes (*p* < 0.05). (**d**) Striatal VMAT2 vesicles associated with the membrane/endosomal fraction had significantly lower dopamine content in METH-treated rats than the saline-treated rats at 1 h after the treatment (−29%). Over the next 24 h, dopamine content decreased to 66%. (**e**) Representative chromatogram for (**d**). * *p* < 0.05, ** *p* < 0.01, *n* = 5–7. Values are expressed as mean ± SEM. Abbreviations: DA, dopamine; METH, methamphetamine; SAL, saline; IP, immunoprecipitation; WB, Western blotting.

We next examined whether there is CDCrel-1: VMAT2 interaction in striatal synaptosomes in the METH-naïve and METH-exposed rats. Anti-VMAT2 antibody immunoprecipitated CDCrel-1 from untreated synaptosomes and METH-treated synaptosomes, with more CDCrel-1 being pulled down at 1 h after METH treatment (Figure 8a,b). Furthermore, there was a significant correlation between CDCrel-1 and VMAT2 immunoreactivity in the membrane/endosomal fraction (Figure 8c).

### 2.9. The Effects of the 4 × 8 mg/kg METH Binge on Dopamine Levels in the Synaptosomal Membrane/Endosomal Fraction

Neurotoxic binge METH largely depletes DA from all VMAT2 vesicle pools in striatal synaptosomes by 1 h. To assess how much DA remained in membrane-bound VMAT2 vesicles, we assessed DA content in synaptosomal membrane/endosomal fractions at 1 h after the last dose of METH or saline. There was a significant main effect of the treatment (METH vs. saline) but no significant main effect of time (1 h vs. 24 h) on DA content in the membrane/endosomal fraction (*F*(1,18) = 22.3, *p* < 0.001, *n* = 5–7, two-way ANOVA with the Holm–Sidak post hoc test). DA content was decreased by 29% and 66% at 1 h and 24 h post-METH, respectively (Figure 8d,e). 

### 2.10. The Effects of the 4 × 8 mg/kg METH Binge on VMAT2-Associated Proteins in the Synaptosomal Membrane/Endosomal Fraction

The intracellular transport of vesicles within terminals is mediated not only by MTs but also by actin microfilaments [53]. Both MTs and actin filaments, as well as filamentous CDCrel-1, play essential roles in the neuronal exocytosis/endocytosis cycle. To examine whether a METH binge alters exocytic and endocytic proteins associated with VMAT2 vesicles, we immunoprecipitated VMAT2 and its interacting partners from membrane/endosomal synaptosomal fractions of saline- and METH-treated rats sacrificed at 1 h after their treatment and analyzed the immunoprecipitated proteins by mass spectrometry (*n* = 4). Among the 1281 proteins detected, an abundance of 10 proteins was significantly altered at FDR < 0.15: 9 increased and 1 decreased (Table 1). Of the 10 proteins, 6 were involved in axonal/intracellular transport and the exocytosis/endocytosis cycle: α-tubulin N-acetyltransferase 1 (ATAT1), protein kinase C, and casein kinase substrate in neurons protein 1 (PACN1, syndapin-1), MT-associated protein RP/EB family member 2 (MARE2), receptor-type tyrosine-protein phosphatase-like N (PTPRN), glia maturation factor beta (GMFB), and sorting nexin-17 (SNX17). Ten differentially expressed proteins are presented in Table 1).

## 3. Discussion

The major findings of this study are as follows: (1) there are wide individual differences in measured indices among outbred Sprague Dawley rats in responses to a 4 × 8 mg/kg METH binge, (2) CDCrel-1 interacts with VMAT2 in vivo and may have a role in VMAT2 trafficking under METH-induced neurotoxicity, (3) parkin does not have a major role in the regulation of VMAT2 or CDCrel-1 levels or trafficking in striatal neuronal terminals, and (4) changes in exocytosis/endocytosis cycle proteins are early responses to the METH binge.

Previous studies have determined that binge administration of high METH doses causes the degeneration of DAergic terminals in the dorsal striatum via oxidative stress, excitotoxicity, and hyperthermia [7]. Hyperthermia is a critical determinant of METH neurotoxicity, with higher temperatures causing more damage [54]. The rats employed in our study were outbred but were of the same strain, sex, and age, and they were housed and assessed under the same conditions. Therefore, the wide individual differences in the thermal and molecular responses to the 4 × 8 mg/kg METH binge were likely due to genetic differences such as variations in genes encoding thermal receptors, antioxidant enzymes, VMAT2, or DA transporters (higher function or levels of DA transporters at the membrane; more METH gets into DAergic terminals). Most studies on METH neurotoxicity used a 4 × 10 mg/kg METH binge. The results from these studies are, for the most part, consistent. The results from this study indicate that individual differences emerge when the sample sizes are sufficiently increased (our sample sizes of the 1 h cohorts were larger than the sample sizes employed in previous studies on METH neurotoxicity). In support of the latter scenario, we found less variability in parkin and CDCrel-1 immunoreactivity in the second experiment, which employed five rats per group (WT vs. PO rat experiment; saline vs. METH). Neuronal responses to oxidative stress and other stressors depend on the severity of the stress. Low-to-moderate oxidative stress increases the levels of proteins involved in stress responses, while severe oxidative stress damages these proteins [55]. Whether stress response proteins are increased or decreased following stress also depends on the length of the post-stress period. The observed U-shape-like responses to METH suggest that, after exposure to the 4 × 8 mg/kg METH binge, some rats could still upregulate their stress response pathways, while some could not.

We previously determined that a 4 × 10 mg/kg METH decreased parkin levels in striatal synaptosomes at 1 h and 24 h after the last dose of the drug, with parkin levels returning to the baseline by 48 h [12]. The decrease was caused by oxidative modification and the degradation of parkin. In this study, 4 × 8 mg/kg METH decreased parkin levels in the total and vesicular/cytosolic synaptosomal fractions at 1 h but not at 24 h after METH. It can be speculated that oxidative stress was less severe in the present study, allowing parkin levels to recover within 24 h. If that was the case, the recovery was likely due to the de novo synthesis of parkin in neuronal perikaryal and axonal transport to the terminals [56]. The discrepancy was more likely due to the different populations of rats used in this study, which expressed more individual differences than those used in the previous study. As with body temperature, high variability in striatal parkin levels was observed in METH-treated rats: a decrease in some rats and no change, or even an increase, in others. Interestingly, significant parkin deficit was detected in rats with low hyperthermia, while highly hyperthermic rats displayed mostly unchanged parkin levels. Hyperthermia itself can cause oxidative stress and protein aggregation [57]. Oxidative stress can decrease the number of viable MTs and inhibit axonal transport [58]. It can also damage degradative systems [59]. Parkin is sensitive to DA-mediated oxidative modifications and prone to misfolding and aggregating under oxidative stress [12,60,61,62,63]. Under conditions of mild oxidative stress, oxidized proteins are rapidly degraded by the 20S proteasome [64,65,66]. When oxidative stress becomes severe, proteasomal function decreases either because of direct oxidative damage, because the accumulation of oxidized proteins exceeds the capacity of proteasomes to clear them, or because proteins are so severely altered that they are no longer recognized as substrates [65,67]. The 26S proteasome plays a predominant role in the normal turnover of parkin, whereas the 20S proteasome and lysosome degrade oxidized and aggregated parkin, respectively [12,60,68]. In view of this knowledge, it might be concluded that oxidatively modified parkin was successfully removed from striatal DAergic terminals in rats with low hyperthermia but not in highly hyperthermic rats, in which severe oxidative stress impaired the proteasome, lysosome, and retrograde axonal transport involved in the removal of damaged proteins from terminals [69]. Another possibility is that oxidative stress changed parkin conformation, obscuring the antibody-binding epitope. Neither explanation agrees with our previous observation of parkin deficit after 4 × 10 mg/kg METH, which would cause more DA-mediated oxidative stress than 4 × 8 mg/kg METH. The deficit suggests viable 20S and lysosomal function and/or MT-mediated axonal transport after 4 × 10 mg/kg METH. A potential explanation of our results is an oxidative stress-independent hyperthermia effect on parkin immunoreactivity.

It has been established that the levels of antioxidant defenses increase in response to mild oxidative stress and decrease in response to severe oxidative stress [70,71,72]. Our finding of increased parkin levels in synaptosomes from METH neurotoxicity-resistant cerebellum agrees with this evidence and suggests that a similar compensatory increase in parkin levels could have taken place in non-DAergic synaptosomes, which are in the majority in striatal synaptosomes, masking parkin deficits in DAergic synaptosomes, and adding to the confounding effect of hyperthermia.

There are three pools of VMAT2 vesicles in the DAergic terminals: (1) the reserve pool, which is away from the active zone at the plasma membrane; (2) the recycling pool that is closer to the active zone; and (3) the readily releasable pool docked and ready for exocytosis [73]. The vesicles are transported into terminals via anterograde axonal transport and can be removed from them via retrograde axonal transport. It has been reported that METH neurotoxicity is associated with impaired VMAT2 trafficking in striatal synaptosomes at 1 h and VMAT2 deficit at 24 h [30,31,47,48]. Specifically, decreases in partially glycosylated VMAT2 were reported in the vesicular/cytosolic fraction at 1 h after 4 × 10 mg/kg METH binge, with membrane/endosomal VMAT2 levels reported to be increased by a study in mice and not changed by a study in rats [30,31]. These results suggest the degradation of VMAT2 or its mobilization to a non-synaptosomal compartment, potentially by the retrograde transport of damaged VMAT2 to the cell bodies in the substantia nigra pars compacta. This study detected a significant decrease in vesicular/cytosolic VMAT2 only at 24 h. Yamamoto’s group reported a decrease in glycosylated VMAT2 in the vesicular/cytosolic fraction at 1 h and it decreased across all synaptosomal fractions at 24 h after 4 × 10 mg/kg METH [47,48]. Given that we used a lower dose of METH binge in this study than that used in the other studies (4 × 8 mg/kg vs. 4 × 10 mg/kg), METH-induced DA-mediated oxidative stress likely needed more time to rise to the threshold necessary to damage VMAT2 and decreased VMAT2 levels in the cytoplasm more than 1 h after the METH binge. Since there was high variability in VMAT2 levels in the METH rats, an alternative explanation for the lack of expected VMAT2 deficit at 1 h is an adaptive increase in VMAT2 levels in some rats and a decrease in others, resulting in a lack of overall change in VMAT2 levels. Despite unchanged levels, VMAT2 function could have been impaired by nitrosylation at 1 h time point in this study. Yamamoto’s group demonstrated the modification of VMAT2 by nitrosylation at 1 h after a 4 × 10 mg/kg METH binge [48]. No significant changes in VMAT2 levels were detected in high-hyperthermia or low-hyperthermia rats. Still, a positive correlation between membrane/endosomal VMAT2 and core body temperature was observed in rats with high hyperthermia, suggesting retention at or mobilization of VMAT2 vesicles to the plasma membrane in high-hyperthermia rats.

Parkin could influence axonal VMAT2 vesicle trafficking by its known interaction with MTs and their stabilization [51]. Moreover, parkin could influence VMAT2 vesicle trafficking between their pools via interactions with α-synuclein [34,74], actin [52], and CDCrel-1, the last of which was reported to be a substrate for parkin in cultured cells [41]. CDCrel-1 is a filamentous protein found attached to membrane and synaptic vesicles [75]. CDCrel-1 binds to syntaxin-1, a component of the soluble NSF attachment protein (SNAP) receptor complex (SNARE), a complex crucial in exocytosis. Through this interaction, CDCrel-1 prevents the docking of vesicles to the membrane and decreases exocytosis [43]. We expected that METH neurotoxicity would decrease parkin levels with a consequent increase in CDCrel-1 levels and an altered distribution of VMAT2 vesicles between the vesicular/cytosolic and membrane/endosomal fractions in striatal synaptosomes. We also expected that parkin overexpression would change VMAT2 distribution between synaptosomal fractions. We found evidence to the contrary, suggesting that parkin does not significantly affect VMAT2 levels or its intracellular trafficking in rat striatal terminals in vivo. Our previous finding of unchanged VMAT2 levels in the METH-naïve striatum in parkin knockout rats supports this conclusion [76].

CDCrel-1 was reported to be a substrate for parkin in vitro [41]. We detected only a small effect of parkin on CDCrel-1 levels in striatal synaptosomes in the METH-naïve rats overexpressing parkin. This finding suggests that parkin is not a major E3 ligase degrading CDCrel-1 in the rat nigrostriatal pathway and agrees with the finding of unchanged CDCrel-1 levels in the striatum of parkin knockout mice [77]. Alternatively, as our synaptosomal preparations contained non-DAergic synaptosomes, which also express CDCrel-1, the effect of parkin overexpression on CDCrel-1 could have been “diluted” in DAergic synaptosomes. The METH binge had a variable effect on striatal synaptosomal CDCrel-1 levels at 1 h. Presently, it is unclear why some rats displayed significant increases in CDCrel-1 levels in striatal synaptosomes while others displayed significant decreases (U-shaped response to METH). This variability did not depend on hyperthermia; however, our correlation analysis showed that high CDCrel-1 levels were accompanied by high parkin levels in METH rats. The METH binge caused a deficit in CDCrel-1 in the WT rats at 1 h, and parkin overexpression appeared to protect vesicular/cytosolic CDCrel-1 from being decreased by METH. These results suggest that parkin is not engaged in the degradation of CDCrel-1 in vivo, but the proteins might interact and that METH damages both proteins via oxidative stress and alters their interaction. At 24 h, CDCrel-1 levels were decreased in the total and vesicular/cytosolic fractions, suggesting the removal of damaged CDCrel-1 from cytosol following the METH binge.

The most apparent interaction found in this study was between CDCrel-1 and VMAT2. This interaction was higher in METH-treated rats than in the saline-treated rats at the 1 h time point and was significant in the membrane/endosomal fraction. CDCrel-1 has been suggested to negatively regulate synaptic vesicle release at presynaptic terminals by forming filamentous barricades at the presynaptic membrane upon interaction with syntaxin-1 [43]. It is possible that, in some rats, METH binge-induced oxidative stress leads to CDCrel-1 accumulation at the plasma membrane, which disables the release of syntaxin-1 and VMAT2 vesicle docking and exocytosis [41]. Such “entrapment” could also prevent the recycling of VMAT2 vesicles and proper sequestration of cytosolic DA.

In summary, multiple toxic effects of METH (hyperthermia, oxidative stress, and excitotoxicity) differently influence the levels and interactions of parkin, VMAT2, and CDCrel-1 in striatal terminals in different rats. This individual variability in responses to binge METH indicates variability in sensitivity to METH neurotoxicity and stress responses in Sprague Dawley rats.

Tubulin and actin are cytoskeletal proteins found in abundance in the cytosol and synaptic active zones, [78] where they are involved in intra-neuronal transport and the exocytosis/endocytosis cycle. The deficits in β-actin immunoreactivity observed in all synaptosomal fractions at 1 h after METH suggest that METH damages actin filaments in striatal terminals. This conclusion is supported by reports of oxidative stress and heat shock inducing the collapse of actin filaments and MTs [79,80]. As parkin binds to both actin filaments and MTs, a deficit in its levels might have contributed to the disorganization of the cytoskeleton in METH binge-treated rats.

β-Actin mediates exocytosis and all kinetically distinguishable forms of endocytosis via membrane pit formation [81,82]. Consequently, METH-induced deficit in β-actin would negatively impact intra-synaptosomal transport as well as the exocytosis/endocytosis cycle of synaptic vesicles, including VMAT2 vesicles. There is evidence for METH altering the exocytosis/endocytosis cycle. Thus, in addition to non-exocytic DA release via the DA transporter, METH upregulates vesicular DA release in the striatum [83,84]. Proteomic/genetic studies have reported altered brain levels of proteins with functions related to the cytoskeleton, transport, endocytosis, and exocytosis after exposure to repeated low and medium METH doses [85,86]. A functional study showed that amphetamines increase endocytosis of the DA transporter [87] and glutamate transporter EAAT3 [88] in DA neurons. Oxidative stress was reported to decrease or increase endocytosis in neurons depending on the stressor and experimental conditions (reviewed in [89]). Finally, our group demonstrated that binge METH administration decreases the function of 26S proteasome and the levels of parkin ([12] and Figure 3a) at 1 h after the last METH injection. Parkin is a component of the ubiquitin-proteasome system. The function of the 26S proteasome is important for exocytosis/endocytosis and DAergic neurotransmission [90], and several parkin substrates are involved in the mediation of exocytosis and/or endocytosis, namely, α-synuclein [34], α- and β-tubulin [35], synaptotagmins IV and 11 [36,37], synphilin-1 [38], endophilin, dynamin, synaptojanin-1 [39], and CDCrel-1 [40,41]. These reports, however, do not provide a complete understanding of METH neurotoxicity-induced alterations in the VMAT2 vesicle exocytosis/endocytosis cycle in DAergic terminals in vivo. Our proteomic data provide more insight into these alterations. The endocytosis-/exocytosis-related parkin substrates mentioned above were found among proteins associated with membrane/endosomal VMAT2; however, their levels were not significantly changed by the METH binge, likely due to the small sample sizes (*n* = 4). Nevertheless, ten proteins were found differentially expressed by the binge METH treatment at 1 h: nine increased and one decreased in abundance. Six of these proteins are involved in axonal/intracellular transport and exocytosis/endocytosis—ATAT1, PACN1, (syndapin-1), MARE2, PTPRN, GMFB (increased), and SNX17 (decreased)—suggesting that the 4 × 8 mg/kg METH binge altered these processes.

ATAT1 localizes to clathrin-coated pits and acetylates α-tubulin. It promotes MT destabilization and accelerates MT dynamics [91]. Tubulin hyperacetylation is associated with cellular responses to stresses, including oxidative stress [92], and increased cell survival through the induction of autophagy [93]. Protein syndapin-1 also decreases MT stability, assists in actin polymerization, and regulates actin cytoskeleton dynamics [94]. Syndapin-1 is required for activity-dependent bulk endocytosis (ADBE) of synaptic vesicles [94]. ADBE generates many synaptic vesicles after intense neuronal activity or increased temperature, replenishing the reserve pool of the vesicles [95,96]. The observed increase in syndapin-1 levels likely occurred in response to METH binge-induced increase in synaptic exocytosis and, therefore, a need for ADBE and new vesicles for the storage of DA released into the cytosol. PTPRN is an important transmembrane protein in dense-core synaptic vesicles (DCVs), involved in cargo loading and the exocytosis of DA in PC12 cells [97]. The function of MARE2 is unknown. The available evidence suggests it may play a role in MT dynamics [98] and axonal delivery of DCVs [99]. The upregulation of PTPRN and MARE2 suggests the mobilization of DCVs to the plasma membrane of DAergic terminals. VMAT2 is associated mainly with small synaptic vesicles but is also found in DCVs [100]. Furthermore, DA was reported to be co-stored with cholecystokinin in DCVs [101]. It is plausible that DCV vesicles were mobilized from substantia nigra pars compacta cell bodies to striatal terminals to increase DA storage by replacing vesicles with dysfunctional nitrosylation-damaged VMAT2. Nexin-17 interacts with many receptors, including integrins, in a sequence-specific manner to regulate their recycling [102,103,104]. Furthermore, nexin-17 participates in the endocytic trafficking and processing of potentially harmful proteins and is linked to autophagy [104]. Autophagy is required to maintain pre-synaptic machinery during neuronal activity under physiological and stressful conditions [105,106]. In presynaptic terminals, the process involves the formation of the autophagosome around the organelle marked for destruction and MT-mediated retrograde transport to cell bodies for fusion with lysosomes and degradation. Nexin-17 is essential for autophagosome component recycling [107]; therefore, the observed downregulation of nexin-17 at 1 h after the METH binge may reflect an impairment of autophagy despite ATAT1 upregulation. In support of this, it has been shown that METH impairs autophagy at several levels, including autophagosome formation and maturation (reviewed in [105]).

FIBA (fibrinogen α-chain) is a blood plasma protein reported to be expressed by astrocytes and neurons under neuroinflammatory or neurodegenerative conditions [108]. FIBA also interacts with its receptors on neurons as well as with integrins and may be involved in axonal repair [108]. For example, FIBA binds to integrin α5β1, which is present on endothelial cells [103,109]. This integrin is involved in inflammatory responses and neuronal regeneration [110]. Another protein involved in responses to stress that was found to increase at 1 h after the METH binge was APOD. APOD is a member of the lipocalin superfamily involved in lipid trafficking, inflammation, and antioxidative responses; it is involved in the axon regeneration process as a lipid transporter [111].

PDK1 and SLC25A42 are mitochondrial proteins. As both mitochondria and synaptic vesicles are attached to actin and plasma membrane, they could have been pulled down by anti-VMAT2 antibody via this connection. PDK1 is a kinase that inhibits the formation of acetyl-coenzyme A and decreases mitochondrial respiration. By this mechanism, it protects cells against oxidative stress and apoptosis [112]. SLC25A42 transports coenzyme A into mitochondria in exchange for ADP. Coenzyme A is necessary for the Krebs cycle function and, consequently, for mitochondria function. Several steps in the exocytosis/endocytosis cycle require ATP, including ADBE, which relies on ATP-dependent actin polymerization [112]. Increases in both PDK1 and SLC25A42 would have opposing effects on mitochondrial function, with a net result being decreased levels of ATP as it was found in deficit at 1 h after binge METH [113]. PDK1 levels likely increased in response to METH-induced oxidative stress and inflammation, while SLC25A42 levels increased to provide ATP for ADBE. Mitochondrial function and ATP levels could also decrease in response to increased nigral neuron firing caused by METH.

GMFB is a protein expressed in glia and some neurons, including substantia nigra neurons [114]. GMFB is upregulated in several neurodegenerative conditions where it predominantly plays detrimental roles [115]. For example, DAergic neurons and astrocytes in GMFβ^−/−^ mice have reduced sensitivity to oxidative stress [116]. On the other hand, overexpression of GFMB increases the expression of the antioxidant enzyme CuZnSOD [115]. GMFB is also a modulator of the actin cytoskeleton [116]. GMFB overexpression reduces the levels of actin-related protein 2/3 complex (Arp2/3), the facilitator of actin polymerization [117]. The increase in GMFB suggests reduced actin polymerization at 1 h after the METH binge, which agrees with previous findings of METH’s ability to modulate actin polymerization and increased actin cycling [50].

Our proteomic data suggest an increased rate of VMAT2 endocytosis/exocytosis cycle in striatal synaptosomes at 1 h after the 4 × 8 mg/kg METH binge, which does not result in significant changes in VMAT2 levels between the studied synaptosomal fractions. VMAT2 cycling could have changed only between the plasma membrane and endosomes. It is also possible that VMAT2 traveled between the terminals and cell body with the rate of its removal equal to its replacement rate. 

Overall, the results suggest that at 1 h after a 4 × 8 mg/kg METH binge, DAergic terminals are engaged in counteracting METH-induced toxic effects, including attempts to increase VMAT2 vesicle endocytosis and autophagy. The levels and trafficking of parkin, VMAT2, and CDCrel-1 are altered differently between the rats, depending on the level of hyperthermia and oxidative stress each animal could combat based on genetic makeup. 

## 4. Materials and Methods

### 4.1. Animals

This study employed young adult male Spraque Dawley rats (~55 days old at the beginning of this study) from Harlan Laboratories (now Envigo, Indianapolis, IN, USA). Upon arrival, the animals were pair-housed and maintained under standard environmental conditions in an AAALAC-accredited vivarium. Thus, the animals were maintained on a 12 h light/dark cycle with continuous ad libitum access to food and water. 

Rats overexpressing parkin in the nigrostriatal dopamine pathway (PO rats) were generated in our laboratory according to a previously published protocol [19]. Briefly, rat parkin-encoding AAV2/6 gene transfer vector (AAV2/6-parkin) was microinjected into the left substantia nigra pars compacta (1 × 10^7^ transduction units); whole non-coding AAV2/6 vector was microinjected into the right one at the following coordinates: −5.6 mm (AP) from Bregma, −2 mm (ML) from Bregma, and −7.6 mm (V) from the dura according to the Paxinos and Watson’s rat brain atlas. After 3 weeks, the rats were treated with binge METH or saline. The non-coding AAV2/6 with a DNA segment cloned upstream of the pgk promoter to adapt the size of vector genome to the AAV packaging capacity (AAV2/6) and rat parkin-encoding AAV2/6 (AAV2/6-parkin) were a kind gift from Dr. Bernard Schneider at the Swiss Federal Institute of Technology Lausanne (EPFL), Switzerland.

A total of 95 rats were used in this study. The first experiment started with 40 rats that were divided into the following four groups based on treatment (METH or saline) and time of sacrifice (1 h or 24 h after the last injection of METH or saline): SAL/1 h, METH/1 h, SAL/24 h, and METH/24 h. More rats were added to the groups because high variability was observed in measured indices after METH administration. A few rats died from METH overdose and were excluded from neurochemical analyses. The final number of rats included in the analyses were as follows: SAL/1 h—*n* = 18, METH/1 h—*n* = 18, SAL/24 h—*n* = 10, and METH/24 h—*n* = 10. The second experiment started with 20 rats. This cohort was divided into METH and saline group. Within each group, some rats were microinjected with adeno-associated viral vector 2/6 coding for parkin (AAV2/6-parkin), some were microinjected with non-coding AAV2/6, and some were microinjected with saline, in the substantia nigra pars compacta. Rats that died following the METH binge were excluded from further analyses. The final number of samples in each subgroup was *n* = 5. To generate tissue for the HPLC and mass spectrometry analyses, 8 more rats were treated with saline and 8 with binge METH. 

### 4.2. Administration of Methamphetamine

(+)-Methamphetamine hydrochloride (METH, 8.0 mg/kg free base) (Sigma-Aldrich, St. Louis, MO, USA) or saline (1 mL/kg) was administered to the rats every 2 h in four successive intraperitoneal (i.p.) injections, ~0.25 mL each. Many previous studies, including ours, have established this treatment paradigm to induce oxidative stress at 1 h and the degeneration of DAergic terminals in rat striatum several days later [7,12]. METH neurotoxicity is associated with hyperthermia, which peaks at approximately 1 h after each injection. Therefore, core body temperatures of the rats were measured via a rectal probe digital thermometer (Thermalert TH-8; Physitemp Instruments, Clifton, NJ, USA) before the beginning of the treatment (baseline temperatures) and at 1 h after each METH or saline injection. Non-anesthetized rats were killed by decapitation at 1 h or 24 h after the last injection of the drug or saline (these time points are commonly used in METH research to assess the short-term effects of binge METH). The brains were removed, and the METH neurotoxicity-sensitive dorsal striatum (referred to hereafter as the striatum) and the control METH neurotoxicity-resistant cerebellum were dissected out, flash-frozen on dry ice, and stored at −80 °C until assayed. The experimental design is presented in Figure 1a.

### 4.3. SDS-PAGE and Western Blotting

Synaptosomal fractions (total synaptosomal, membrane/endosomal, and vesicular/cytosolic) were prepared from striatal and cerebellar tissue by differential centrifugation as previously described [19]. Specifically, tissue pieces were homogenized in 0.5 mL 0.32 M sucrose with protease inhibitor cocktail and centrifuged at 800× *g* for 24 min at 4 °C. The supernatant was then centrifuged at 22,000× *g* for 17 min at 4 °C. The pellet was resuspended in 150 µL ice-cold distilled water and retained as the total synaptosomal fraction. Part of the total synaptosomal fraction was further centrifuged at 22,000× *g* for 17 min at 4 °C, and the supernatant was retained as the vesicular/cytosolic fraction, while the pellet was resuspended in 150 µL ice-cold distilled water and retained as the membrane/endosomal fraction. Sample protein concentrations were determined using a Bradford protein assay, using bovine serum albumin as the standard.

Synaptosomal fractions were subjected to reducing sodium dodecyl sulfate-polyacrylamide gel electrophoresis (SDS–PAGE). The 10–20 μg proteins per lane were loaded on 4–12% Bis-Tris gels (Life Technologies, Grand Island, NY, USA). Electrophoresis and Western blotting were performed as previously described [76], utilizing the following primary antibodies: CDCrel-1 (1:1000, overnight at 4 °C) (MAB5358; Chemicon International, Temecula, CA, USA); parkin (1:1000; overnight at 4 °C) (Prk8; Cell Signaling Technology, Danvers, MA, USA); β-actin (1:1000; 1 h at room temperature) (8H10D10; Signaling Technology, Danvers, MA, USA); α-tubulin (1:1000, overnight at 4 °C) (sc58668; Santa Cruz Biotech, Santa Cruz, CA, USA), and VMAT2 (1:3000, overnight at 4 °C) (NBP1-69750H, Novus Biologicals, Toronto, ON, Canada), as well as appropriate secondary antibodies. Blots were developed using ECL detection and an LAS4000 bioimager (GE Healthcare, Piscataway, NJ, USA). Immunoreactivity was quantified using ImageJ software v.1.50 (National Institutes of Health, Bethesda, MD, USA). For standardization across the blots, each blot contained all experimental groups. The Western blot data were expressed as relative optical density units and normalized to the controls on each blot. This approach normalized differences in the development of the blot and across blots.

### 4.4. Immunohistochemistry

Midbrains were post-fixed with 4% paraformaldehyde, frozen in isopentane, and kept at −80 °C for further immunohistochemistry procedures. Coronal brain sections (30 μm thick) containing substantia nigra pars compacta were sliced on a cryostat (Thermo Fisher Waltham, MA, USA) and processed as previously described [19]. Citrate buffer antigen retrieval (ThermoFisher, Waltham, MA, USA) was applied to all tissue sections. The sections were incubated overnight at 4 °C with the anti-parkin antibody (Prk8; Cell Signaling Technology) and antibody against DAergic marker tyrosine hydroxylase) (AB152, EMD Millipore Corp., Billerica, MA, USA) both diluted 1:100 in the blocking buffer. The sections were then incubated for 2.5 h at room temperature with Alexa Fluor-488 conjugated secondary antibody (Invitrogen, Carlsbad, CA, USA). DRAQ5 (Invitrogen) was used to stain nuclei. The sections were then mounted using Fluoromount mounting medium (Southern Biotech, Birmingham, AL, USA). The immunostaining on each slice (3 sections per slice) was imaged using the Leica TCS SPE-II laser scanning confocal microscope (Leica, Wetzlar, Germany) and averaged per rat.

### 4.5. Co-Immunoprecipitation

Striatal synaptosomal fractions were prepared from rats euthanized at 1 h after the METH binge. Dynabeads (Life Technologies, Grand Island, NY, USA) were incubated with 2 μL of either the rabbit polyclonal VMAT2 primary antibody (NBP1-69750H, Novus Biologicals), mouse monoclonal CDCrel-1 primary antibody (MAB5358; Chemicon International, Temecula, CA, USA), parkin mouse monoclonal primary antibody (Prk8, 1:1000; Cell Signaling Technology), or radioimmunoprecipitation assay (RIPA) buffer (negative control) for 6–12 h at 4 °C. This was followed by the addition of synaptosomal fractions (100–200 µg) and a second round of incubation (12 h at 4 °C). For all quantitative coimmunoprecipitation studies, equal protein content of the synaptosomal fraction was incubated with beads for each representing group (saline or METH). Following each incubation, the beads were washed three times using phosphate-buffered saline containing 0.02% Tween-20 (PBST). Following immunoprecipitation, parkin-, CDCrel-1-, and VMAT2-associated proteins were separated from the beads using SDS Tris–Glycine sample buffer (Bio-Rad, Hercules, CA, USA) and heating (10 min at 70 °C). The supernatants were run on 4–12% Bis-Tris gels under reducing conditions and subjected to Western blot analysis using the mouse CDCrel-1 primary antibody and anti-mouse secondary antibody, as described above.

### 4.6. Mass Spectrometry

Membrane/endosomal fractions of synaptosomes were incubated with Dynabeads conjugated with anti-VMAT2 antibody and processed as described for coimmunoprecipitation. The resulting eluted samples were washed and subjected to SDS-PAGE. After staining with Sypro Ruby stain to visualize proteins, multiple gel pieces were excised, washed with 25 mM NH_4_HCO_3_/50% acetonitrile (for 15 min with each solution), dehydrated in 100% acetonitrile (ACN), rehydrated in 50 mM NH_4_HCO_3,_ and dehydrated again in 100% ACN. Subsequently, the gel pieces were vacuum-dried for 5 min. Next, the following reactions were performed: reduction with 5 mM dithiothreitol in 50 mM NH_4_HCO_3_ and alkylation with 15 mM iodoacetic acid in 50 mM NH_4_HCO_3_. The gel pieces were again dehydrated with ACN, the solvent was removed, and sequencing-grade trypsin (Promega, Madison, WI, USA) in 25 mM NH_4_HCO_3_/10% ACN was added to rehydrate the gel slices and initiate overnight digestion. Following digestion, peptides were extracted from the gel plugs using 50% ACN/0.05% formic acid. The free peptides were then speed-vacuumed to dryness and solubilized in 2% ACN/0.1% formic acid.

The peptides were separated by reverse phase chromatography (Acclaim PepMap RSLC C18 column, Thermo Scientific, Waltham, MA, USA), followed by ionization with the Nanospray Flex Ion Source (Thermo Scientific), and introduced into a Q Exactive mass spectrometer (Thermo Scientific). Abundant species were fragmented with high-energy collision-induced dissociation (HCD). Data analysis was performed using Proteome Discoverer 1.4 (Thermo Fisher, Waltham, MA, USA), which was incorporated with the Mascot algorithm (Matrix Science, Boston, MA, USA). The Swiss Prot rat protein database downloaded on March 2013 was used, and a reverse decoy protein database was run simultaneously for false discovery rate (FDR) determination. Secondary analysis was performed using Scaffold 4.2.1 (Proteome Software, Portland, OR, USA). The minimum protein identification probability was set at ≥95% with 2 unique peptides at ≥99% minimum peptide identification probability. Only those proteins identified with at least 2 unique peptides, a *p* < 0.05, a Benjamin–Hochberg FDR < 0.15, and a fold change >1.5 were considered differentially expressed.

### 4.7. High-Performance Liquid Chromatography

The synaptosomal membrane/endosomal pellet was obtained as described in Chu et al. [118]. Briefly, striatal tissue was homogenized in ice-cold 0.32 M sucrose solution. The homogenate was centrifuged at 800× *g* for 24 min at 4 °C. The supernatant was centrifuged at 22,000× *g* for 17 min at 4 °C to obtain the synaptosomal pellet. The pellet was resuspended in 75 μl of ice-cold water, and osmolarity was immediately restored by adding an equal volume of pH 7.5 buffer containing 25 mM HEPES and 100 mM potassium tartrate. The resulting solution was fractionated at 22,000× *g* for 17 min at 4 °C into the membrane/endosomal and vesicular/cytosolic fractions. The membrane/endosomal pellet was resuspended in ice-cold perchloric acid (final concentration 0.3 N). The resulting solution was then centrifuged at 22,000× *g* for 30 min 4 °C to obtain the precipitated protein pellet. The supernatant was analyzed for DA content using high-performance liquid chromatography (HPLC), using a Shimadzu Prominence HPLC system with electrochemical detection (Shimadzu Scientific Instruments, Columbia, MD, USA) as published previously [12]. Samples (20 μL) were injected onto a 3 μm C-18 reverse phase column (150 × 3.2 mm, 3 μM particle size, Thermo Scientific). DA was eluted with a mobile phase consisting of 90 mM sodium dihydrogen phosphate monohydrate, 50 mM citric acid, 1.7 mM 1-octane sulfonic acid, 50 μM EDTA, and 10% acetonitrile (pH 3.8). The analytes were detected using an electrochemical detector (Dionex Coulochem III, E1 = −150 mV, E2 = +220 mV). The protein pellet was resuspended in 1 N NaOH overnight at 4 °C, and its concentration was determined using the Bradford assay. To determine DA content, 20 μL of the supernatant was injected into a C-18 reverse phase column (ThermoScientific). The mobile phase consisted of 50 mM sodium citrate, 50 mM sodium phosphate, 200 μM EDTA, 1.5 mM heptane sulphonic acid, and 14% methanol adjusted to pH 3.8. The concentration of DA was quantified by interpolating peak areas relative to those generated by a range of appropriate standards (Sigma Aldrich, St. Louis, MO, USA). The DA values were normalized to protein content in each sample and expressed as pg of analyte per μg of protein.

### 4.8. Statistical Analyses

Statistical analyses were performed using the program GraphPad Prism (GraphPad Software, version 10.3.1, San Diego, CA, USA). The comparisons made in this study were pre-planned. We established a priori that the striatum was the METH-affected brain region while the cerebellum was unaffected by METH based on existing knowledge of the effects of binge METH in the rat brain. Based on previously published results, samples were assumed to come from populations with the same standard deviations. Two-way or mixed model (if values were missing) repeated measures (RT) ANOVA followed by the Sidak post hoc test was performed on body core temperature data. The Greenhouse–Geisser correction was used if there was a lack of sphericity. Differences between the control and METH groups were analyzed separately in each synaptosomal fraction by multiple unpaired *t*-tests and the Holm–Sidak method to correct for multiple comparisons. Two-way ANOVA followed by the Holm–Sidak post hoc test was employed when the data had two variables. Correlations were determined using simple regression analysis and Pearson’s correlation test. The Western blotting data were expressed as relative optical density units on each gel normalized to the controls. This approach normalized differences across blots, allowing standardization across the treatment groups. The data are presented as mean ± standard error (SEM). Statistical significance was set at *p* < 0.05.

## 5. Limitations of This Study

There are several limitations of this study. The sample sizes were small in the second (PO vs. WT rats) and third (HPLC, DA content) experiments. Given the individual differences that emerged in the first experiment, substantially increasing the sizes of all experimental groups would provide more information on individual responses to METH neurotoxicity. Oxidative stress indices were not measured. An assessment of the severity of oxidative stress in each experimental animal would provide information on whether it was a factor driving individual differences in responses to the METH binge. This study was largely observational and did not establish causal relationships between the measured variables. Another limitation of our research is that a mix of DAergic and non-DAergic synaptosomes was assessed. However, this was because the population of DAergic synaptosomes in the striatum constitutes <1% of the overall synaptosomal population and is difficult to separate. The presence of non-DAergic terminals (mostly glutamatergic) introduced a confounder and complicated data interpretation.

## 6. Conclusions and Future Directions

The major conclusion of this study is that studying large groups of outbred rats such as Sprague Dawley rats can help define individual molecular and genetic differences in responses to a neurotoxic METH binge. Our research shows, for the first time, that individual differences include the trafficking of VMAT2 and CDCrel-1 in striatal terminals as well as core body temperature. Our results also indicate that a neurotoxic METH binge induces changes to multiple proteins involved in the exocytosis/endocytosis cycle in striatal, most likely DAergic, terminals. This has not been shown before. Finally, we demonstrate that outbred rats, such as Sprague Dawley rats, can serve as a model for studying individual differences in responses to METH and other neurotoxins.

Future experiments should employ large sample sizes in longitudinal studies to delineate the subgroups differing in responses to METH neurotoxicity. Adding more time points will reveal changes over time, providing a more comprehensive understanding of striatal terminal responses to METH neurotoxicity. Future genetic and proteomic analyses of these responses will shed light on molecular mechanisms driving these responses. Variations in genes encoding thermal receptors, antioxidant enzymes, VMAT2, or the DA transporter should be of particular focus. The incorporation of experimental designs for establishing causal relationships between variables should follow. Establishing a causal relationship between genetic makeup and individual ability to handle METH-induced stresses in rodents will aid the development of individual treatment strategies for humans suffering from METH use disorder and its neurological consequences.

## Figures and Tables

**Table 1:** List of differentially changed (FDR < 0.15, fold change > 1.5) proteins after binge METH treatment.

Accession #	Protein Symbol	Protein Name	Function	Fold Change
Q6MG11	ATAT_RAT	Alpha-tubulin N-acetyltransferase 1	Acetylates α-tubulin on MTs, promotes MT destabilization, and accelerates MT dynamics	9.2
Q9Z0W5	PACN1_RAT	Protein kinase C and casein kinase substrate in neurons protein 1	Reorganizes MT and actin cytoskeleton, decreases MT polymerization and stability, and is required for bulk endocytosis	2.7
Q3B8Q0	MARE2_RAT	Microtubule-associated protein RP/EB family member 2	Unknown; may be involved in MT polymerization and dynamics	1.9
Q63259	PTPRN_RAT	Receptor-type tyrosine-protein phosphatase-like N	Neurotransmitter loading into dense-core synaptic vesicles	2.4
Q63228	GMFB_RAT	Glia maturation factor beta	Role in the growth, differentiation, and stress responses in neurons and glia, and actin-mediated endocytosis	11
Q6AYS6	SNX17_RAT	Sorting nexin-17	Regulates endocytic trafficking of several proteins, plays a role in protein sorting and autophagy	0.45
Q63065	PDK1_RAT	Pyruvate dehydrogenase (acetyl-transferring) kinase isozyme 1, mitochondrial	Regulation of glucose and fatty acid metabolism, and responses to cellular stresses	3.3
P0C546	S2542_RAT	Mitochondrial coenzyme A transporter SLC25A42	Transports coenzyme A into mitochondria in exchange for ADP	2.0
P06399	FIBA_RAT	Fibrinogen alpha chain	Role in responses to injury and inflammation, and axonal repair	2.5
P23593	APOD_RAT	Apolipoprotein D	Role in lipid trafficking, food intake, inflammation, antioxidative responses, and axon regeneration	5.8

## Data Availability

Raw data and western blots are available from the corresponding author upon request. The original or representative blots have been provided to the publisher.

## Supplementary Materials

The following supporting information can be downloaded at https://www.mdpi.com/article/10.3390/ijms252313070/s1.
