# Supplementary material for: Interactions of VMAT2 with CDCrel-1 and Parkin in Methamphetamine Neurotoxicity
Source: Int J Mol Sci. 2024 Dec 5;25(23):13070. doi: 10.3390/ijms252313070 (PMC11642102; doi:10.3390/ijms252313070)
Supplement: Supplementary file 1 [file ijms-25-13070-s001.zip › ijms-3200269-supplementary.pdf]

Supplementary Materials

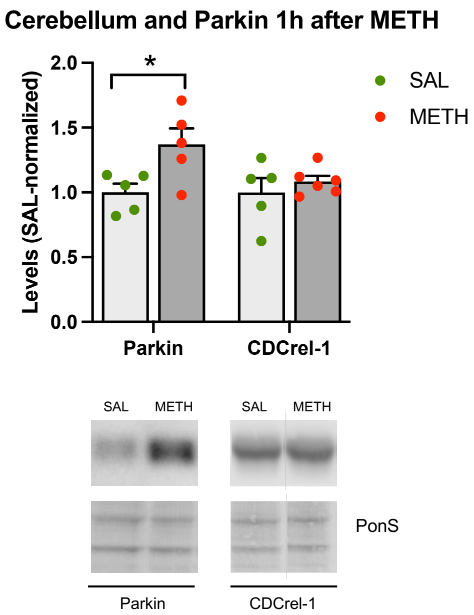

Figure S1. The effects of 4x8mg/kg METH binge on parkin and CDCrel-1 immunoreactivity in cerebellar synaptosomes. Immunoreactivity of parkin (left) and CDCrel-1 (right) in cerebellar synaptosomes in saline- and METH-treated rats at 1h after the treatment. **p*<0.05. Vertical grey lines show where the blot was cut for rearrangement. Values are expressed as mean ± SEM. Abbreviations: METH, methamphetamine; SAL, saline; PonS, Ponceau S.

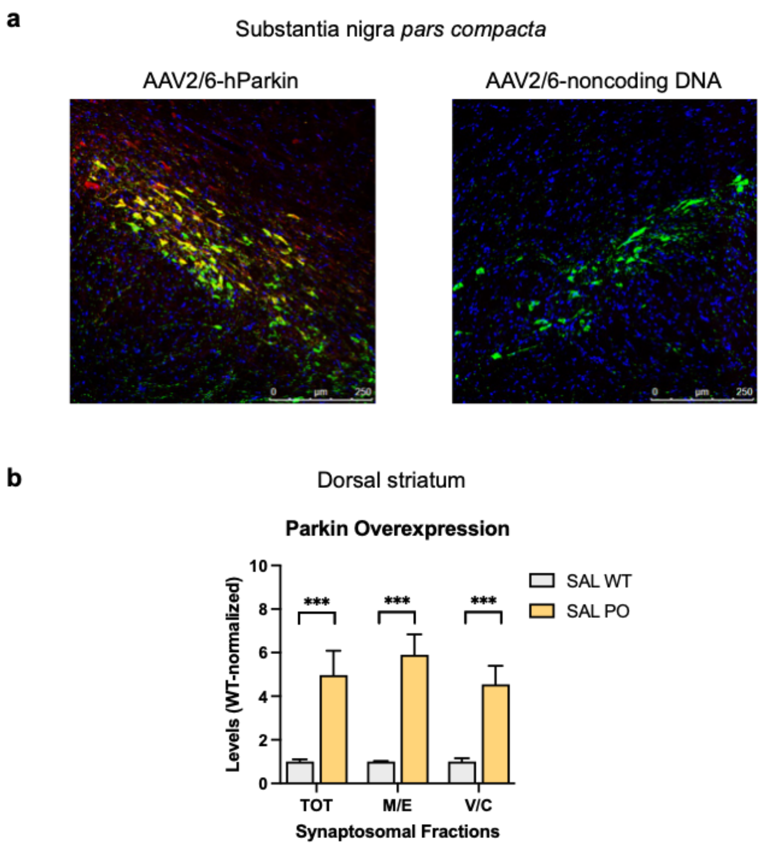

Figure S2. Validation of parkin overexpression in the substantia nigra pars compacta and dorsal striatum. (a) The left SNpc was microinjected with parkin-encoding AAV2/6 gene transfer vector (AAV2/6-parkin) whereas the right substantia nigra was microinjected with non-coding AAV2/6. Immunoreactivity of parkin (red fluorescence) and dopaminergic marker tyrosine hydroxylase (green fluorescence) in the left and right substantia nigra *pars compacta* (SNpc). The yellow color indicates parkin immunofluorescence overlapping with tyrosine hydroxylase fluorescence, thus confirming parkin overexpression in the left SNpc. Parkin is at very low levels in the nigrostriatal pathway; therefore, it is hard to detect by immunofluorescence in wild-type rats. Bars: 200µm. **(b)** Parkin immunoreactivity (chemiluminescence) increased ~5-fold in all synaptosomal fractions in the left dorsal striatum after microinjection of the AAV2/6-parkin into the left SNc. ***$p<0.001$. Abbreviations: METH, methamphetamine; SAL, saline.

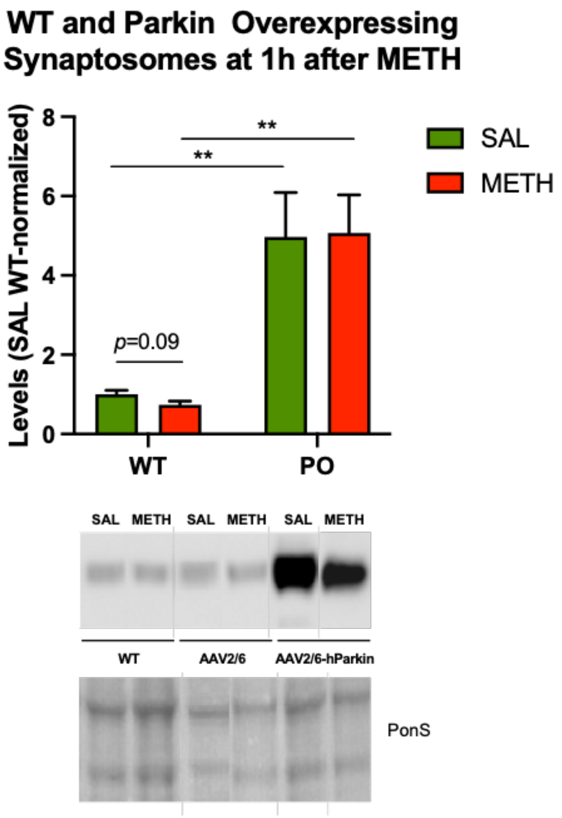

Figure S3. The effects of 4x8mg/kg METH binge on parkin immunoreactivity in striatal synaptosomal fractions. Figure 5b (top) shows parkin immunoreactivity in the total synaptosomal fraction in wild-type and parkin-overexpressing rats treated with saline or binge METH. At the bottom are representative blots and Ponceau loading control staining. ***p*<0.01. Vertical grey lines show where the blot was cut for rearrangement. Abbreviations: METH, methamphetamine; SAL, saline.
